# Planned morning aerobic exercise in a fasted state increases energy intake in the preceding 24 h

**DOI:** 10.1007/s00394-021-02501-7

**Published:** 2021-02-23

**Authors:** Asya Barutcu, Elizabeth Briasco, Jake Moon, David J. Stensel, James A. King, Gemma L. Witcomb, Lewis J. James

**Affiliations:** School of Sport, Exercise and Health Sciences, Loughborough University, Leicestershire, LE11 3TU UK

**Keywords:** Appetite, Energy intake, Eating behavior, Weight loss, Exercise

## Abstract

**Purpose:**

We previously observed increased energy intake (EI) at the meal before planned afternoon exercise, but the proximity of the meal to exercise might have reduced the scale of the pre-exercise anticipatory eating. Therefore, this study examined EI in the 24 h before fasted morning exercise.

**Methods:**

Fourteen males, experienced with gym-based aerobic exercise (age 25 ± 5 years, BMI 23.8 ± 2.5 kg/m^2^), completed counterbalanced exercise (EX) and resting (REST) trials. On day 1, subjects were told the following morning’s activity (EX/REST), before eating ad-libitum laboratory-based breakfast and lunch meals and a home-based afternoon/evening food pack. The following morning, subjects completed 30-min cycling and 30-min running (EX; 3274 ± 278 kJ) or 60-min supine rest (REST; 311 ± 34 kJ) fasted. Appetite was measured periodically, and EI quantified.

**Results:**

Afternoon/evening EI (EX 7371 ± 2176 kJ; REST 6437 ± 2070 kJ; *P* = 0.017) and total 24-h EI (EX 14,055 ± 3672 kJ; REST 12,718 ± 3379 kJ; *P* = 0.011) were greater during EX, with no difference between trials at breakfast (*P* = 0.761) or lunch (*P* = 0.071). Relative EI (EI minus energy expended through EX/REST) was lower in EX (EX 10,781 ± 3539 kJ; REST 12,407 ± 3385 kJ; *P* = 0.004).

**Conclusion:**

This study suggests planned fasted aerobic exercise increases EI during the preceding afternoon/evening, precipitating a ~ 10% increase in EI in the preceding 24-h. However, this increase did not fully compensate for energy expended during exercise; meaning exercise induced an acute negative energy balance.

## Introduction

As the prevalence of overweight and obesity continues to rise globally, effective strategies to facilitate successful weight control are needed [1]. Prevention and management of obesity rely on the components of behaviour that influence energy balance, including physical activity [2]. In basic terms, energy balance is determined as energy intake (in consumed food and drink) minus energy expenditure (through metabolic processes and activity). Therefore, theoretically, individuals can either reduce energy consumed in food and drink or increase activity to disrupt energy balance and induce weight loss or prevent weight gain [3]. However, in practice this interaction is very complex and influenced by a multitude of factors, making successful weight control difficult [4–6].

Aerobic exercise influences physiological components of appetite regulation [2, 7, 8]. A substantial amount of research has now been conducted on the acute effects of exercise on appetite and energy intake; generally reporting that acute aerobic exercise does not alter energy intake in the hours afterwards, compared to a resting control condition [9]. Although it is worth noting that research investigating higher exercise intensities (i.e. > 70% *V*O_2max_) consistently demonstrates a reduction in perceived hunger, but this does not always translate to similar changes in energy intake [10–13]. Therefore, in the acute setting, relative energy intake (energy consumed minus energy expended through exercise/rest) is reduced, resulting in a short term energy deficit [14]. In theory, this acute energy deficit should result in weight loss due to the disparity in energy balance. Many exercise intervention studies have tested this theory, in an attempt to induce weight loss (e.g. [15–19]). However, results from these studies have shown that there is considerable interindividual variability and the expected reductions in weight (calculated from exercise energy expenditure) are often not induced. Several mechanisms, such as compensatory increases in hunger/energy intake [20] and decreases in non-exercise physical activity [21] have been proposed to explain this response. However, evidence now suggests that non-exercise physical activity and resting metabolic rate are unaffected by exercise training [22], therefore shifting the focus towards the other side of the energy balance equation (i.e. energy intake).

Roughly 60 years ago, Jean Mayer suggested the concept that exercise induces compensatory increases in energy intake to restore energy balance [23]. This concept, however intuitive, is not consistent with short-term energy intake measurements made at meals consumed post-exercise, where exercise seems not to affect energy intake [9, 24]. Whilst exercise appears to induce changes in hormones secreted from the gastrointestinal tract (e.g. ghrelin, peptide tyrosine tyrosine, etc.), these endocrine alterations with exercise do not appear to influence energy intake, at least acutely [9]. More recently, studies have started to examine eating behaviour in the pre-exercise period [25–27]. In contrast to previous work examining energy intake after aerobic exercise, Barutcu, Witcomb and James [26] reported that when a post-exercise meal is planned in advance of aerobic exercise, energy intake was ~ 24% greater than for planned rest. Similarly, other studies have shown that ad-libitum energy intake is increased when snack items [25] or meals [27] are provided before impending aerobic exercise compared to resting conditions.

One consideration with these previous studies is that the proximity of the pre-exercise snack/meal to the exercise session may have tempered the responses observed [25, 27]. Given the short time interval between the pre-exercise meal and the exercise session (1–3 h) in these previous studies [25, 27], it is possible that subjects individual considerations about the effect of food intake on gastrointestinal comfort during exercise may have influenced their desire/motivation to increase energy intake. Although many exercise sessions will take place in the immediate post-prandial period, many will not, including sessions taking place after an overnight fast. Therefore, this study investigated the effect of an anticipated exercise session in the morning after a > 9 h fast (to remove any potential effects of proximity of exercise to meals on eating behaviour) on appetite and energy intake in the preceding 24 h and compared these responses to an identical resting control trial. It was hypothesised that energy intake over the afternoon/evening meal, and perhaps lunch (given the results from our previous work), but not breakfast, would be greater before exercise compared to rest.

## Methods

### Subjects

Subjects were fourteen healthy, non-smoking, weight stable (self-reported), and habitually active (< 10 h per week) males, (age 25 ± 5 years; BMI 23.8 ± 2.5 kg/m^2^; body fat % 17.2 ± 4.4; *V*O_2_max 47.6 ± 4.0 ml kg min^−1^). Subjects provided written consent before completing the study. Ethical approval was obtained from the Loughborough University Ethics Approvals (Human Participants) Sub Committee (reference number: R17-P133), but as this was not a clinical trial, it was not pre-registered on a clinical trials database. Subjects were not taking any medications known to affect appetite, and were also not restricted, disinhibited, or hungry eaters (i.e. not in the clinical range), as determined by the Three-Factor Eating Questionnaire [28]. Each subject completed two preliminary trials and two experimental trials in a randomised counterbalanced order. Using the data from a previous study from our laboratory [27], an alpha of 0.05, a beta of 0.8 and a between trial correlation of 0.8, it was determined that 14 subjects would be required to detect a 15% difference in total 24 h energy intake between trials.

### Pre-trial standardisation

In the 24 h preceding the first experimental trial, subjects recorded their dietary intake and physical activity. These diet and activity patterns were replicated before the second experimental trial and adherence to these requirements was verbally confirmed before trials. Strenuous exercise and alcohol intake were also not permitted during this period.

### Preliminary trials

Subjects completed two preliminary trials. During the first trial, which took place at a time convenient for the subject, height (to nearest 0.1 cm; SECA stadiometer, Germany), and body mass (to nearest 0.01 kg; Adam Equipment, CFM-150 scales, UK) were measured. Subcutaneous body fat was estimated from skinfold measurements at four sites (biceps, triceps, supra-iliac, sub-scapula) using callipers using callipers (Harpenden, UK). The sum of all four sites were used to estimate body density [29] and percentage of body fat [30]. Subjects also completed questionnaires to assess health status and eating patterns and performed two submaximal tests: one on a cycle ergometer (Lode Corival, Groningen, Holland) and one on a motorised treadmill (h/p/cosmos sports and medical GmbH/Munich, Germany). Sub-maximal tests involved four, 4-min stages of workloads between 80 and 280 W (cycle ergometer) and 6–13 km/h (treadmill), with each stage separated by a short rest period. At the end of each stage, heart rate (Polar M400, Kempele, Finland) and rating of perceived exertion (RPE) [31] were recorded.

After a short break, subjects then completed a maximal incremental exercise test to exhaustion (*V*O_2_peak) on the treadmill, commencing at a gradient of 1% and at a speed estimated to elicit a heart rate of ~ 160 beats/min. The gradient increased by 1% every minute until volitional exhaustion. Expired gas was collected during the final minute of this maximal incremental exercise test and heart rate and RPE were recorded every minute. Intensities for the exercise session during experimental trials were derived from the heart rate-exercise intensity relationship determined in the sub-maximal stages, as well as the peak heart rate reached during the *V*O_2_peak test.

During the second preliminary trial, subjects arrived at the laboratory at 0800 h and were asked to complete a visual analogue scale for subjective appetite ratings (detailed below). Subjects then started the exercise session in a fasted state to ensure they were experienced with the specific exercise session and the laboratory environment. The exercise session involved a total of 60 min exercise (30 min cycling and 30 min running), as described in the exercise trial. An ad-libitum buffet breakfast was served 30-min post-exercise, and subjects were free to leave after this period. Visual analogue scales were completed throughout the day. Three hours post-breakfast, subjects arrived back at the laboratory for lunch and were provided with visual analogue scales for the rest of the day, as well as an afternoon/evening food pack. Subjects were not allowed to eat for 2 h post-lunch and after 2300 h in the evening.

### Experimental trials

Subjects completed two, 2-day experimental trials; exercise (EX) and rest (REST) in a randomised counter-balanced order and separated by at least 4 days.

On day 1, subjects arrived at the laboratory at 0800 h for breakfast. A baseline appetite questionnaire was completed upon arrival (0800 h) and post-void body mass was recorded in light clothing. Subjects were then told if they were on the EX or REST trial (i.e. what they would do the following morning) and given 30 min to consume breakfast (0800–0830 h) from a selection of cold ready-to-eat foods. Upon completion of breakfast, subjects completed another appetite questionnaire (0830 h) and left the laboratory, before returning at 1200 h for lunch. Lunch was served for 30 min and consisted of a buffet selection of ready-to-eat cold foods. Appetite questionnaires were completed before and after lunch. After lunch, subjects were provided with a food pack containing a main evening meal and a selection of snack items from which they were free to consume during the afternoon/evening period. The entire food pack (including all empty wrappers) was returned to the laboratory the following morning. Standard instructions were read to subjects before the breakfast and lunch buffet meals on day 1. These were as follows: “You have 30 min to have your breakfast. Remember that you are on the EX/REST trial tomorrow, so please choose your food items accordingly. You are welcome to eat whatever and however much you want from the selection. If you want more of anything, please let the researchers know and we will put out more food”. For the evening meal “You are not allowed to eat anything for 2 h after lunch, after this time you are free to eat whatever and however much you want from the food pack provided until 2300 h. You are not allowed anything else that is not in the pack apart from water. When you’re ready to have your pasta meal, please use the bowl provided and put any leftovers back in the Tupperware box. Please keep all wrappers and fruit skins and return everything the next day”.

On day 2, subjects returned to the laboratory at 0800 h and completed another appetite questionnaire, before post-void body mass was again measured, and they completed the 60-min exercise/rest. In the EX trial, exercise consisted of 30 min of steady-state cycling at a workload equal to ~ 75% HR-max and 30 min of steady state running at a running speed equal to ~ 80% HR-max. Heart rate was recorded every 5 min and RPE was recorded every 15 min during exercise. Expired gas samples were collected between 14–15 min and 29–30 min during cycling and running. All expired gas samples collected in experimental trials were analysed for oxygen and carbon dioxide concentration (Servomex 1440, Crowborough, UK), volume (Harvard Dry Gas Meter, Harvard Ltd, Edenbridge, UK) and temperature (Edale, Cambridge, UK). Ambient room air was analysed concurrent with expired gas collections to correct *V*O_2_ and *V*CO_2_ values [32].

For the REST trial, day 1 was the same as described above. On day 2, instead of completing the exercise session, subjects rested on a portable bed in a supine position for 60 min, with expired gas samples collected at 25–30 min and 55–60 min.

### Study foods

For all meals, foods were provided in excess of expected consumption. Breakfast and lunch were multi-item buffet meals, consisting of cold ready-to-eat foods, which were presented in a research kitchen (Table 1). Subjects chose foods from the buffet and ate in isolation in a separate dining room, returning to the buffet as many times as they desired during each 30-min meal period. For both breakfast and lunch, the amount consumed was quantified by weighing the food before and after consumption, with macronutrient content of foods ascertained from manufacturer values.

**Table 1:** Items provided at each meal

Breakfast buffet items		
White bread	Cornflakes—cereal	Peanut butter spread
Brown bread	Weetabix—cereal	Nutella spread
Rice Krispies—cereal	Strawberry yoghurt	Strawberry jam spread
Crunchy nut—cereal	Raspberry yoghurt	Bananas
Shreddies—cereal	Cherry yoghurt	Apples
Coco pops—cereal	Apple juice	Clementines
Cheerios—cereal	Orange juice	Milk
Lunch buffet items		
White bread	Cherry yoghurt	Salt and vinegar crisps
Brown bread	Strawberry yoghurt	Cheese and onion crisps
Mature cheddar cheese	Raspberry yoghurt	Orange squash
Honey smoked ham	Cadbury mini rolls	Summer fruits squash
Grilled chicken pieces	Mayonnaise	Apples
Can of tuna	Butter	Clementines
Lettuce	Chocolate chip cookies	
Tomatoes	Salted crisps	
Evening meal		
Nutri-grain apple cereal bar	Mini cookies	Clementines
Nutri-grain blueberry cereal bar	Salted crisps	Bananas
Nutri-grain strawberry cereal bar	Salt and vinegar crisps	Strawberry yoghurt
Mars chocolate—fun size	Cheese and onion crisps	Cherry yoghurt
Twix chocolate—fun size	Prawn cocktail crisp	Raspberry yoghurt
Maltesers chocolate—fun size	Apples	Tomato pasta meal

The afternoon/evening food pack contained a homogenous cheese and tomato pasta meal (6.60 ± 0.05 kJ g^−1^, with 14%, 60%, 25% and 1% of the energy provided by protein, carbohydrate, fat and fibre, respectively) prepared in a standard manner the day before the experimental trial and given to the subjects cold. Additional food consisting of a selection of confectionary snacks, cereal bars, crisps, yogurts, etc. (Table 1) were supplied in the pack. Subjects were free to consume what they wanted from the food pack at any time between 1430 and 2300 h. The pack, as well as any empty wrappers, Tupperware, fruit skins/cores and leftovers were returned the following morning. Energy intake was determined by weighing food items before and after they were returned the next day, with macronutrient content of foods ascertained from manufacturer values. Food was not allowed in the 2 h post-lunch and after 2300 h.

### Subjective appetite sensations

Subjects rated their feelings of hunger ‘How hungry do you feel?’, fullness ‘How full do you feel?’, desire to eat (DTE) ‘How strong is your desire to eat?’, and prospective food consumption (PFC) ‘How much food do you think you could eat?’ on 100 mm visual analogue scales throughout the day [33]. Verbal anchors of ‘not at all’/’none at all’/’no desire at all’ and ‘extremely’/’a lot’ were placed at 0 and 100 mm, respectively. Appetite was rated at 0800 upon arrival at the lab on both days, as well as before and after each meal and before bed on day 1, and at the mid-point and end of exercise/rest on day 2.

### Statistical analysis

Data were analysed using SPSS 23.0 (SPSS Inc., Somers, NY, USA). All data were checked for normality of distribution using a Shapiro–Wilk test. Data containing one factor were analysed using a *t* test or Wilcoxon signed-rank test, as appropriate. Data containing two factors were analysed using a two-way repeated measures ANOVA and, where appropriate, followed by Bonferroni-adjusted paired *t* tests or Bonferroni-adjusted Wilcoxon signed-rank tests, as appropriate. Data sets were determined to be significantly different when *P* ≤ 0.05. Data are presented as mean ± standard deviation throughout, unless otherwise stated.

## Results

### Pre-trial measures

Pre-trial (i.e. day 1) body mass (EX 78.7 ± 6.5 kg; REST 78.4 ± 6.7 kg; *t* = 1.193; *df* = 13; *P* = 0.254), hunger (*Z* = − 0.534; *P* = 0.594), fullness (*Z* = − 0.196; *P* = 0.844), DTE (*t* = 0.582; *df *= 13; *P* = 0.571) and PFC (*t* = 0.583; *df* = 13; *P* = 0.570) did not differ between trials.

### Energy and macronutrient intake

**Fig. 1:**
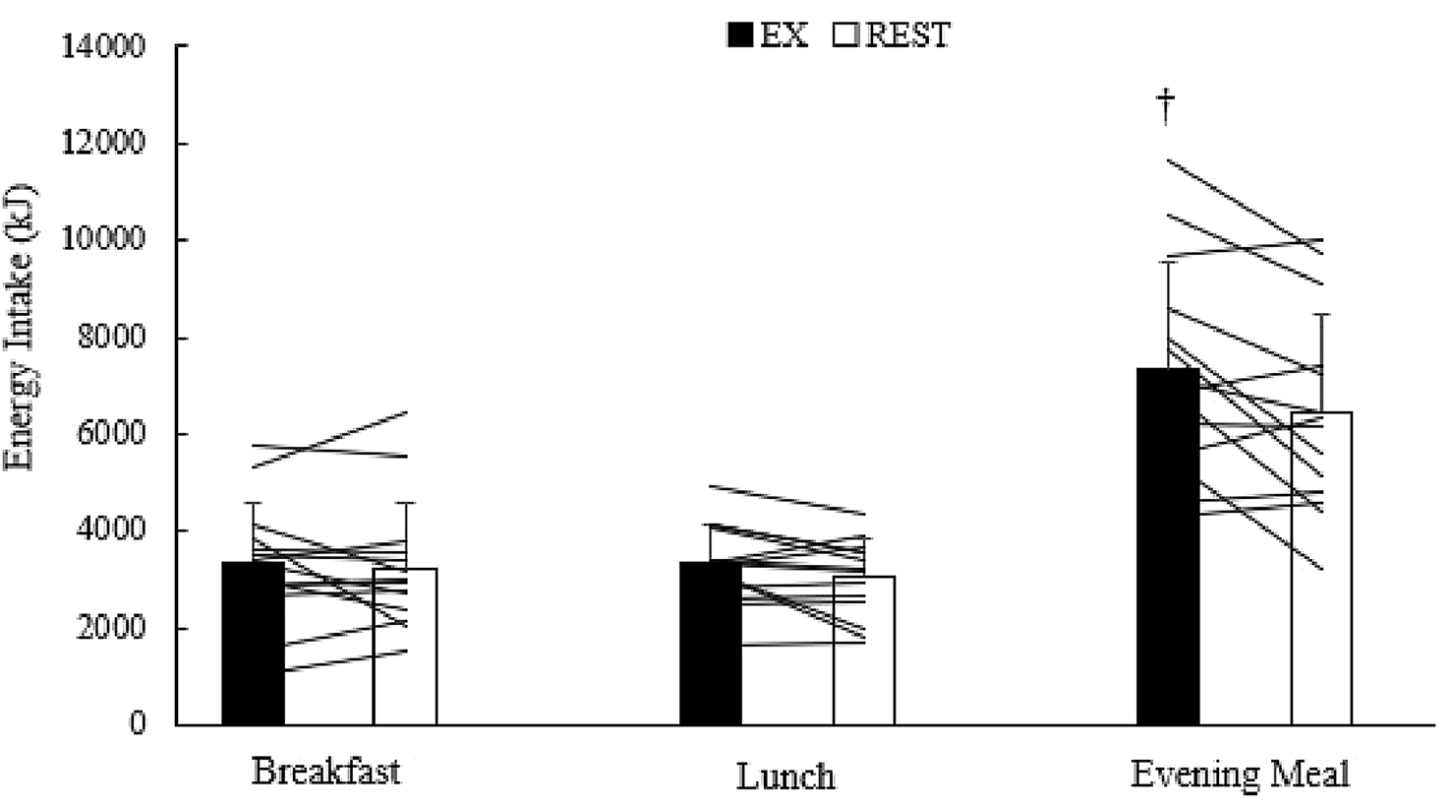
Breakfast, lunch and evening meal energy intakes during both trials. Values are mean ± SD. Lines that intersect the EX and REST bars represent individual data. ^†^Indicates values significantly different to REST (*P* < 0.05)

**Fig. 2:**
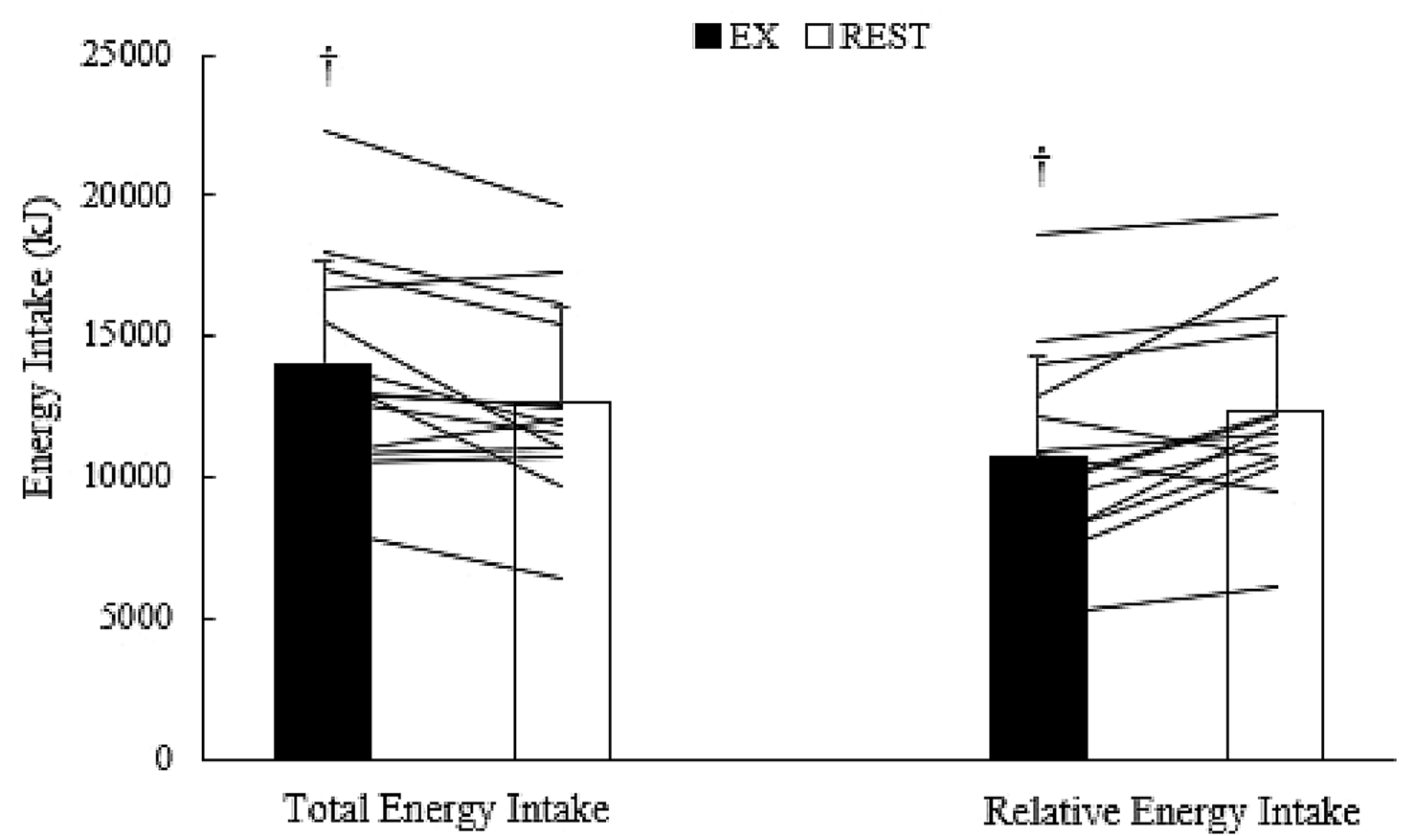
Total energy intake and Relative energy intake values for both trials. Values are mean ± SD. Lines that intersect the EX and REST bars represent individual data. ^†^Indicates values significantly different to REST (*P* < 0.05)

On day 1, energy intake at breakfast (*Z* = − 0.345; *P* = 0.761) and lunch (*t* = 1.964; *df* = 13; *P* = 0.071) were not different between trials, although the data for lunch approached significance. However, energy intake over the evening was ~ 930 kJ (~ 13%) greater in EX compared to REST (*t* = 2.723; *df* = 13; *P* = 0.017) (Table 2; Fig. 1). Total 24-h energy intake on day 1 was ~ 1340 kJ (~ 10%) greater in EX compared to REST (*t* = 2.966; *df* = 13; *P* = 0.011) (Table 2; Fig. 2).

**Table 2:** Total energy (kJ), carbohydrate (CHO), protein (PRO), fat, and fibre intakes on day 1 for each meal during both trials

	Total energy (kJ)	CHO (g)	PRO (g)	FAT (g)	Fibre (g)
Breakfast					
EX	3358 ± 1254	129.9 ± 47.2	26.6 ± 10.3	17.7 ± 12.1	8.6 ± 4.2
REST	3248 ± 1337	126.6 ± 41.8	26.4 ± 10.9	16.5 ± 13.0	8.1 ± 4.6
Lunch					
EX	3326 ± 817	71.3 ± 19.8	45.4 ± 15.7^a^	34.7 ± 14.7	7.7 ± 2.0
REST	3033 ± 803	63.3 ± 22.3	42.0 ± 14.8	32.2 ± 12.6	7.1 ± 2.1
Afternoon/evening period					
EX	7371 ± 2176^a^	258.8 ± 77.4^a^	47.2 ± 11.7^a^	55.2 ± 20.1^a^	11.1 ± 4.2^a^
REST	6437 ± 2070	228.1 ± 71.7	40.9 ± 11.2	47.8 ± 18.4	9.9 ± 3.9
Total 24 h					
EX	14,055 ± 3672^a^	460.0 ± 125.3^a^	119.4 ± 23.9^a^	107.6 ± 36.4^a^	27.4 ± 8.2
REST	12,718 ± 3379	418.0 ± 104.4	109.3 ± 23.0	96.5 ± 36.6	25.1 ± 6.5

Data are mean ± SD

^a^Indicates values significantly different to REST (*P* < 0.05). Please note that the evening pasta meal was homogenous in nature; therefore; macronutrient intake is proportional to volume consumed

On day 2, energy expenditure during the 60-min exercise/rest session was ~ 2960 kJ greater during EX (EX 3274 ± 278 kJ; REST 311 ± 34 kJ; *t* = 38.887; *df *= 13; *P* < 0.001) and, therefore, relative energy intake (energy intake on day 1 minus energy expended during exercise/rest) was less in EX (*t* = − 3.451; *df* = 13; *P* = 0.004).

Regarding macronutrient intakes, protein intake was greater in EX at lunch (*t* = 2.228; *df* = 13; *P* = 0.044), whilst intake of all macronutrients was greater in EX the afternoon/evening (*P* ≤ 0.042). Over the 24-h pre-exercise, intakes of carbohydrate (+ 10%; *P* = 0.025), protein (+ 9%; *P* = 0.017) and fat (+ 11%; *P* = 0.005) were all greater in EX (Table 2).

### Steady state exercise measures

All mean steady state values (HR, RPE, *V*O_2_, RER) are presented in Table 3. *V*O_2_ (*t* = 34.751; *df* = 13; *P* < 0.0001) and RER (*t* = 3.120; *df* = 13; *P* = 0.009) were greater during EX.

**Table 3:** *V*O_2,_ RER, heart rate and RPE during EX and REST trials

	*V*O_2_ (L/min)	RER	Heart rate (bpm)	RPE
EX	2.51 ± 0.213^a^	0.95 ± 0.033^a^	143 ± 21	13 ± 1
REST	0.25 ± 0.003	0.85 ± 0.03	N/A	N/A

Data are mean ± SD

^a^Indicates values significantly different to REST (*P* < 0.05)

### Subjective appetite sensations

**Fig. 3:**
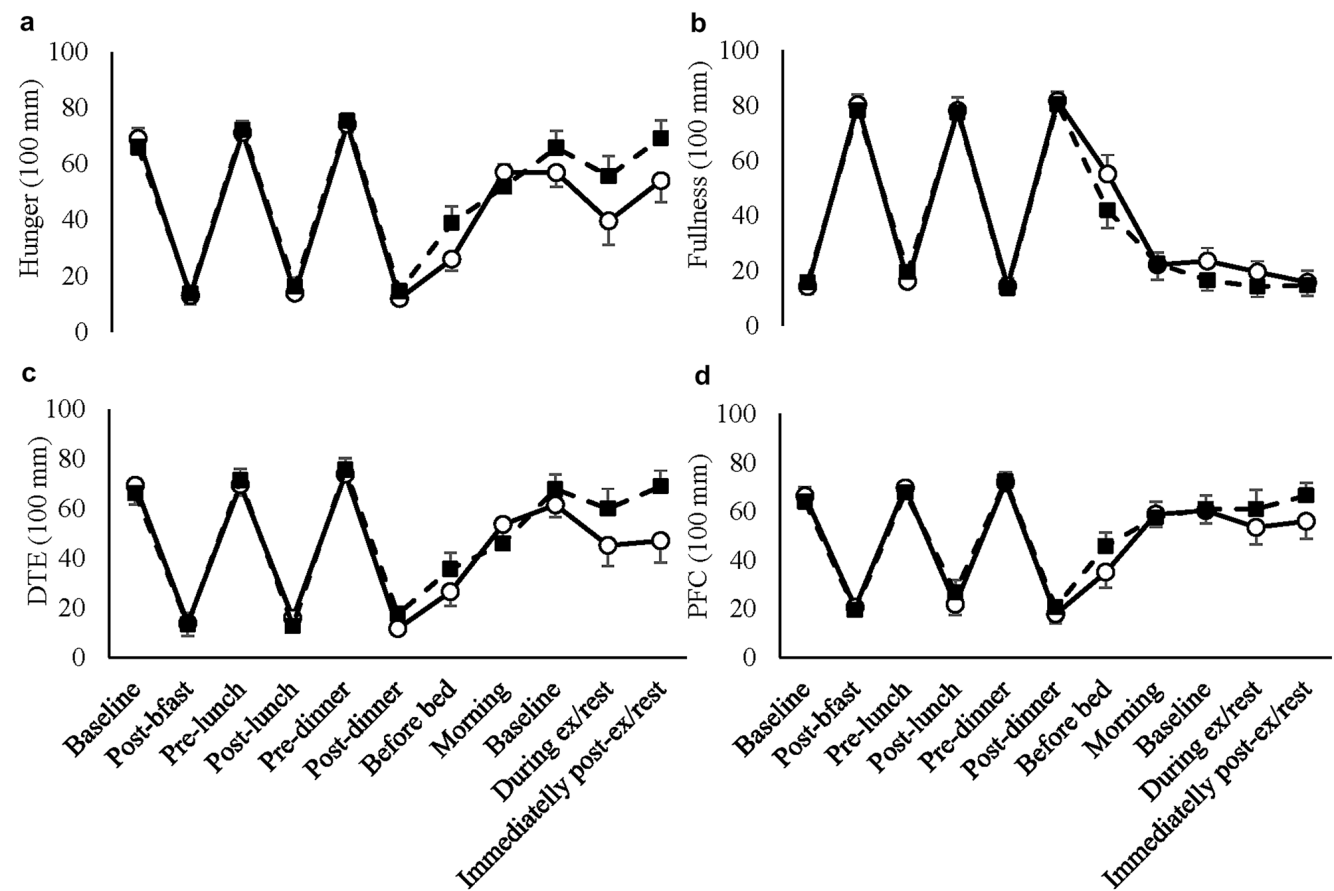
Hunger (**a**), fullness (**b**), DTE (**c**) and PFC (**d**) ratings for day 1. The solid line presents EX and dotted line presents REST trial. Values are mean ± SEM. ^†^Indicates significance

There were main trial effects for hunger (*F*(*df* = 1) = 13.257; *P* = 0.003), desire to eat (*F*(1) = 14.060; *P* = 0.002) and prospective food consumption (*F*(*df* = 1) = 7.338; *P* = 0.018), but not fullness (*F*(*df* = 1) = 3.606; *P* = 0.080). There were no time × trial interaction effects for any of the subjective appetite ratings (hunger: *F*(*df* = 4.485) = 2.217; *P* = 0.071; fullness: *F*(*df* = 3.914) = 1.039; *P* = 0.396; DTE: *F*(*df* = 4.416) = 1.942; *P* = 0.109; PFC: *F*(*df *= 4.526) = 1.238; *P* = 0.304; Fig. 3). There were main effects of time for all subjective appetite ratings (*P* < 0.001).

## Discussion

This study investigated the effect of an anticipated morning-fasted aerobic exercise session on appetite and energy intake in the 24 h before exercise, compared to an identical resting control trial. It was hypothesised that energy intake over the afternoon/evening, and potentially at lunch, but not at breakfast, would be greater for exercise compared to rest. In line with this hypothesis, energy intake during the afternoon/evening was ~ 13% greater in EX compared to REST, whilst energy intake at breakfast and lunch were not significantly different between trials.

These results are consistent with the findings of two previous studies that have examined eating behaviour at meals served before known exercise sessions [25, 27]. Results from most previous studies investigating the effects of acute exercise on appetite and energy intake suggest that energy intake after exercise is no different to after a similar period of rest [9, 10]. Interestingly, aerobic exercise appears to produce endocrine effects that one might hypothesise would decrease post-exercise energy intake. Exercise decreases concentrations of acylated ghrelin [34, 35] and increases concentrations of peptide tyrosine tyrosine (PYY) [36]. These effects should, in theory, result in reduced appetite and energy intake at the subsequent meal according to ghrelin’s orexigenic [37] and PYY’s anorexigenic characteristics [38]. Whilst reductions in subjective appetite are commonly reported during or immediately after exercise [39, 40], these reductions are usually corrected shortly after exercise has ceased and before eating, but endocrine alterations can persist [41]. Whilst aerobic exercise does, on the whole, produce weight loss, the typical weight loss observed is far from what would be predicted on the basis of the energy deficit created by acute studies [42–44]. In the present study, exercise still reduced relative energy intake, but the increase in pre-exercise energy intake was sufficient to compensate for ~ 45% of the energy deficit created. This is a substantial compensation and might, at least partially, account for the less than expected weight loss observed in previous exercise training studies.

This eating behaviour has also been observed in another recent study that investigated the effects of a future exercise session on pre-exercise energy intake in sedentary overweight males [25]. In this study, subjects (restrained and unrestrained) were provided with a standardised breakfast and lunch followed by an ad-libitum snack (potato chips) 60-min before a known exercise or rest session. Unlike the present study, where all subjects completed a standardised 60-min aerobic exercise session, subjects were instructed to complete an exercise session until they considered they had completed a “sufficient workout”. This study reported that restrained eaters consumed significantly more (~ 677 kJ) before the exercise trial compared to a resting trial: however, this effect was not present with the unrestrained eaters. In contrast to the results from Sim et al*.* [25], the present study observed that unrestrained eaters increased their energy intake by ~ 1337 kJ the day before a morning exercise session. This may be explained by the differences in eating opportunities provided, as in the current study subjects had access to ad-libitum breakfast, lunch and evening meals in the 24 h pre-exercise, consisting of a variety of food items, whereas, Sim et al. [25] provided only one *ad-libitum* eating opportunity 60 min before exercise, with only one food option (potato chips). Furthermore, given that the subjects of Sim et al*.* [25] were sedentary individuals, as opposed to the regular exercisers in the present study, their lack of experience with exercise training may have reduced their tendency to supplement their energy intake in anticipation of exercise.

In this context, there is evidence to suggest that responses for energy intake in the hours after exercise are different between active and inactive individuals [9]. According to the meta-analysis of Schubert et al*.* [9], the absolute energy intake values obtained after an exercise session indicated that individuals who engaged in lower levels of physical activity or exercise were more receptive to the appetite suppressing effects of exercise. This is also supported by the work of Jokisch et al*.* [45] and Mayer and Thomas [46]. These studies suggest that there may be some conditioning, whereby regular exercisers may learn to increase their energy intake around exercise over time. Given that expected satiety/satiation characteristics of a food are strong predictors of portion size selection, which are also learnt from previous experiences [47–49], it could be argued that decisions about food intake in close temporal proximity to exercise may also be influenced by previous experiences of exercise (specifically energy expenditure). Speculatively, this conditioning might play a part in determining a portion size in the hours before (or indeed after) an exercise session. In line with this, McCaig et al*.* [50] observed that subjects increased energy intake at the subsequent test meal when they were informed that they had expended ~ 1110 kJ, compared to a group who were informed that they expended ~ 210 kJ. Indeed, the study of McCaig et al*.* [50] adds weight to the hypothesis that the anticipation of an exercise session, or energy expenditure per se, may influence decisions about meal planning, and subsequently portion size/food consumption. Taken together, and whilst speculative, results from these studies may clarify some of the less than expected weight loss observed in chronic exercise intervention studies [51] and future studies should examine this element of eating behaviour in chronic exercise training studies.

Other possible mechanisms that could explain the results from this study could be the Compensatory Health Beliefs Model [52]. The Compensatory Health Beliefs Model suggests that certain unhealthy behaviours (i.e. having extra energy/food) can be compensated for by engaging in positive, healthy behaviours (such as exercise) [52, 53]. This model could partially explain the results of the present study as the subjects may have justified, to themselves, that the upcoming exercise session provided them with the ‘license’ to eat more food in advance of the exercise. Initially, we observed this behaviour in our previous work [27] where compensation occurred only at the preceding mealtime before exercise, however we were uncertain how far in advance this compensatory behaviour would come into effect given the lack of research in this area. The prospect of completing a 60-min aerobic exercise session after a > 9 h fast may have also encouraged subjects to consume more during the evening meal in the hope that they would have enough energy available to carry out the task. Additionally, recommendations given to athletes suggest increasing energy, especially carbohydrate, intake in the hours before exercise [54]. However, given that such recommendations for athletes infiltrate into lay publications (e.g. magazines and online resources), regular exercisers may develop the view that they too should also increase their energy intake. Energy intake was only increased in EX in the afternoon/evening, which combined with our previous work [27], suggests that it may only be the meal that immediately precedes exercise where additional energy is ingested. Future work should focus on exploring these effects over multiple sessions, as well as exploring the interaction between pre-and post-exercise energy compensation in situations were individuals are exercising on a regular (perhaps daily) basis, as in this setting one sessions post-exercise nutrition may also be the next session’s pre-exercise nutrition.

It appears, from the results of this and previous studies [25–27], that when exercisers make decisions about food intake in advance of an impending exercise session, their energy intake/portion size might be increased. Clearly the weight status or, perhaps more importantly, the weight management goals of the subjects might have played a part in their decision (conscious or unconscious) to increase energy intake in advance of exercise. The subjects in the present study were weight stable, not trying to lose weight, and habitually physically active. Therefore, they would be achieving energy balance on a day-to-day basis, meaning it is logical that they would increase their energy intake where energy expenditure is increased. Thus, it would be of great importance to understand whether this phenomenon of increasing energy intake in anticipation of an exercise session is apparent in overweight/obese populations where weight loss is a goal, and this should be the focus of future work in this area. What accounts for this effect is not known and should be the focus of future studies as it may help to reduce compensatory eating and strengthen the energy deficit created by exercise. Although speculative, it would be interesting to know if it was caused by a ‘licencing effect’, where individuals might have perceived the impending energy expenditure through exercise to licence them to increase their EI.

In conclusion, this study demonstrates that anticipation of fasted, morning aerobic exercise may cause individuals to increase their energy intake the evening before. Whilst this increase in pre-exercise energy intake compensated for 45% of the energy deficit created by exercise, the exercise session still produced an acute negative energy balance. The results from this study better our understanding of the relationship between exercise and energy intake/balance and suggest a potential time period where those engaged with chronic exercise training might increase their energy intake. These results might help to explain, at least partially, why aerobic exercise training studies often do not observe the anticipated weight loss with training. Long-term exercise studies that employ inactive individuals might go some way to explaining the mechanisms and timescales behind this phenomenon.
